# Supplementary material for: Mapping the evolution of fertility support policies in China: A content and instrumental analysis
Source: PLoS One. 2025 Oct 9;20(10):e0332137. doi: 10.1371/journal.pone.0332137 (PMC12510515; doi:10.1371/journal.pone.0332137)
Supplement: S1 Appendix — (ZIP) [file pone.0332137.s001.zip › S1 Appendix. 226 original policy documents/52-国务院办公厅关于促进养老托育服务健康发展的意见(FBM-CLI-2-349820).doc]

国务院办公厅关于促进养老托育服务健康发展的意见

**发布部门：** [国务院办公厅](https://www.pkulaw.com/law/chl?Aggs.IssueDepartment=601) [机构沿革](javascript:void(0))

**发文字号：**国办发〔2020〕52号

**发布日期：**2020.12.14

**实施日期：**2020.12.14

**时效性：** [现行有效](https://www.pkulaw.com/law/chl?Aggs.TimelinessDic=01)

**效力级别：** [国务院规范性文件](https://www.pkulaw.com/law/chl?Aggs.EffectivenessDic=XC0203)

**法规类别：** [社会福利与社会保障](https://www.pkulaw.com/law/chl?Aggs.Category=01505) [老少妇幼残保护](https://www.pkulaw.com/law/chl?Aggs.Category=01604)

国务院办公厅关于促进养老托育服务健康发展的意见
（国办发〔2020〕52号）

各省、自治区、直辖市人民政府，国务院各部委、各直属机构：
　　促进养老托育服务健康发展，有利于改善民生福祉，有利于促进家庭和谐，有利于培育经济发展新动能。为贯彻落实党中央、国务院决策部署，更好发挥各级政府作用，更充分激发社会力量活力，更好实现社会效益和经济效益相统一，持续提高人民群众的获得感、幸福感、安全感，经国务院同意，现提出以下意见。
　　**一、**健全老有所养、幼有所育的政策体系
　　（一）分层次加强科学规划布局。根据“一老一小”人口分布和结构变化，科学谋划“十四五”养老托育服务体系，促进服务能力提质扩容和区域均衡布局。省级人民政府要将养老托育纳入国民经济和社会发展规划统筹推进，并制定“十四五”养老托育专项规划或实施方案。建立常态化督查机制，督促专项规划或实施方案的编制和实施，确保新建住宅小区与配套养老托育服务设施同步规划、同步建设、同步验收、同步交付。
　　（二）统筹推进城乡养老托育发展。强化政府保基本兜底线职能，健全基本养老服务体系。优化乡村养老设施布局，整合区域内服务资源，开展社会化管理运营，不断拓展乡镇敬老院服务能力和辐射范围。完善老年人助餐服务体系，加强农村老年餐桌建设。探索在脱贫地区和城镇流动人口集聚区设置活动培训场所，依托基层力量提供集中托育、育儿指导、养护培训等服务，加强婴幼儿身心健康、社会交往、认知水平等方面早期发展干预。
　　（三）积极支持普惠性服务发展。大力发展成本可负担、方便可及的普惠性养老托育服务。引导各类主体提供普惠性服务，支持非营利性机构发展，综合运用规划、土地、住房、财政、投资、融资、人才等支持政策，扩大服务供给，提高服务质量，提升可持续发展能力。优化养老托育营商环境，推进要素市场制度建设，实现要素价格市场决定、流动自主有序、配置高效公平，促进公平竞争。
　　（四）强化用地保障和存量资源利用。在年度建设用地供应计划中保障养老托育用地需求，并结合实际安排在合理区位。调整优化并适当放宽土地和规划要求，支持各类主体利用存量低效用地和商业服务用地等开展养老托育服务。在不违反国家强制性标准和规定前提下，各地可结合实际制定存量房屋和设施改造为养老托育场所设施的建设标准、指南和实施办法。建立健全“一事一议”机制，定期集中处置存量房屋和设施改造手续办理、邻避民扰等问题。在城市居住社区建设补短板和城镇老旧小区改造中统筹推进养老托育服务设施建设，鼓励地方探索将老旧小区中的国企房屋和设施以适当方式转交政府集中改造利用。支持在社区综合服务设施开辟空间用于“一老一小”服务，探索允许空置公租房免费提供给社会力量供其在社区为老年人开展助餐助行、日间照料、康复护理、老年教育等服务。支持将各类房屋和设施用于发展养老托育，鼓励适当放宽最长租赁期限。非独立场所按照相关安全标准改造建设托育点并通过验收的，不需变更土地和房屋性质。
　　（五）推动财税支持政策落地。各地要建立工作协同机制，加强部门信息互通共享，确保税费优惠政策全面、及时惠及市场主体。同步考虑公建服务设施建设与后期运营保障，加强项目支出规划管理。完善运营补贴激励机制，引导养老服务机构优先接收经济困难的失能失智、高龄、计划生育特殊家庭老年人。对吸纳符合条件劳动者的养老托育机构按规定给予社保补贴。
　　（六）提高人才要素供给能力。加强老年医学、老年护理、社会工作、婴幼儿发展与健康管理、婴幼儿保育等学科专业建设，结合行业发展动态优化专业设置，完善教学标准，加大培养力度。按照国家职业技能标准和行业企业评价规范，加强养老托育从业人员岗前培训、岗位技能提升培训、转岗转业培训和创业培训。加大脱贫地区相关技能培训力度，推动大城市养老托育服务需求与脱贫地区劳动力供给有效对接。深化校企合作，培育产教融合型企业，支持实训基地建设，推行养老托育“职业培训包”和“工学一体化”培训模式。

　　**二、**扩大多方参与、多种方式的服务供给
　　（七）增强家庭照护能力。支持优质机构、行业协会开发公益课程，利用互联网平台等免费开放，依托居委会、村委会等基层力量提供养老育幼家庭指导服务，帮助家庭成员提高照护能力。建立常态化指导监督机制，加强政策宣传引导，强化家庭赡养老年人和监护婴幼儿的主体责任，落实监护人对孤寡老人、遗弃儿童的监护责任。
　　（八）优化居家社区服务。发展集中管理运营的社区养老和托育服务网络，支持具备综合功能的社区服务设施建设，引导专业化机构进社区、进家庭。建立家庭托育点登记备案制度，研究出台家庭托育点管理办法，明确登记管理、人员资质、服务规模、监督管理等制度规范，鼓励开展互助式服务。
　　（九）提升公办机构服务水平。加强公办和公建民营养老机构建设，坚持公益属性，切实满足特困人员集中供养需求。建立入住综合评估制度，结合服务能力适当拓展服务对象，重点为经济困难的失能失智、高龄、计划生育特殊家庭老年人提供托养服务。完善公建民营机制，打破以价格为主的筛选标准，综合从业信誉、服务水平、可持续性等质量指标，引进养老托育运营机构早期介入、全程参与项目工程建设，探索开展连锁化运营。
　　（十）推动培训疗养资源转型发展养老服务。按照“应改尽改、能转则转”的原则，将转型发展养老服务作为党政机关和国有企事业单位所属培训疗养机构改革的主要方向。各地要加大政策支持和协调推进力度，集中解决资产划转、改变土地用途、房屋报建、规划衔接等困难，确保转养老服务项目2022年底前基本投入运营。鼓励培训疗养资源丰富、养老需求较大的中东部地区先行突破，重点推进。
　　（十一）拓宽普惠性服务供给渠道。实施普惠养老托育专项行动，发挥中央预算内投资引领作用，以投资换机制，引导地方政府制定支持性“政策包”，带动企业提供普惠性“服务包”，建设一批普惠性养老服务机构和托育服务机构。推动有条件的用人单位以单独或联合相关单位共同举办的方式，在工作场所为职工提供托育服务。支持大型园区建设服务区内员工的托育设施。
　　（十二）引导金融机构提升服务质效。鼓励政府出资产业投资基金及市场化的创业投资基金、私募股权基金等按照市场化、法治化原则，加大对养老托育领域的投资力度。创新信贷支持方式，在依法合规、风险可控、商业可持续前提下，推进应收账款质押贷款，探索收费权质押贷款，落实好信贷人员尽职免责政策。鼓励金融机构合理确定贷款期限，灵活提供循环贷款、年审制贷款、分期还本付息等多种贷款产品和服务。扩大实施养老产业专项企业债券和养老项目收益债券，支持合理灵活设置债券期限、选择权及还本付息方式，鼓励发行可续期债券。引导保险等金融机构探索开发有针对性的金融产品，向养老托育行业提供增信支持。支持保险机构开发相关责任险及养老托育机构运营相关保险。

　　**三、**打造创新融合、包容开放的发展环境
　　（十三）促进康养融合发展。支持面向老年人的健康管理、预防干预、养生保健、健身休闲、文化娱乐、旅居养老等业态深度融合。发挥中医药独特优势，促进中医药资源广泛服务老年人群体。支持各类机构举办老年大学、参与老年教育，推动举办“老年开放大学”、“网上老年大学”，搭建全国老年教育资源共享和公共服务平台。
　　（十四）深化医养有机结合。发展养老服务联合体，支持根据老年人健康状况在居家、社区、机构间接续养老。为居家老年人提供上门医疗卫生服务，构建失能老年人长期照护服务体系。有效利用社区卫生服务机构、乡镇卫生院等基层医疗资源，开展社区医养结合能力提升行动。针对公共卫生突发事件，提升养老机构应急保障能力，增设隔离功能并配备必要的防控物资和设备，加强工作人员应急知识培训。
　　（十五）强化产品研发和创新设计。健全以企业为主体的创新体系，鼓励采用新技术、新工艺、新材料、新装备，增强以质量和信誉为核心的品牌意识，建立健全企业知识产权管理体系，推进高价值专利培育和商标品牌建设，培育养老托育服务、乳粉奶业、动画设计与制作等行业民族品牌。促进“一老一小”用品制造业设计能力提升，完善创新设计生态系统。
　　（十六）促进用品制造提质升级。逐步完善养老托育服务和相关用品标准体系，加强标准制修订，强化标准实施推广，探索建立老年用品认证制度。推进互联网、大数据、人工智能、5G等信息技术和智能硬件的深度应用，促进养老托育用品制造向智能制造、柔性生产等数字化方式转型。推进智能服务机器人后发赶超，启动康复辅助器具应用推广工程，实施智慧老龄化技术推广应用工程，构建安全便捷的智能化养老基础设施体系。鼓励国内外多方共建养老托育产业合作园区，加强市场、规则、标准方面的软联通，打造制造业创新示范高地。
　　（十七）培育智慧养老托育新业态。创新发展健康咨询、紧急救护、慢性病管理、生活照护、物品代购等智慧健康养老服务。发展“互联网＋养老服务”，充分考虑老年群体使用感受，研究开发适老化智能产品，简化应用程序使用步骤及操作界面，引导帮助老年人融入信息化社会，创新“子女网上下单、老人体验服务”等消费模式，鼓励大型互联网企业全面对接养老服务需求，支持优质养老机构平台化发展，培育区域性、行业性综合信息平台。发展互联网直播互动式家庭育儿服务，鼓励开发婴幼儿养育课程、父母课堂等。
　　（十八）加强宜居环境建设。普及公共基础设施无障碍建设，鼓励有条件的地区结合城镇老旧小区改造加装电梯。加强母婴设施配套，在具备条件的公共场所普遍设置专席及绿色通道。引导房地产项目开发充分考虑养老育幼需求。指导各地加快推进老年人居家适老化改造。以满足老年人生活需求和营造婴幼儿成长环境为导向，推动形成一批具有示范意义的活力发展城市和社区。

　　**四、**完善依法从严、便利高效的监管服务
　　（十九）完善养老托育服务综合监管体系。以养老托育机构质量安全、从业人员、运营秩序等方面为重点加强监管。落实政府在制度建设、行业规划、行政执法等方面的监管责任，实行监管清单式管理，明确监管事项、监管依据、监管措施、监管流程，监管结果及时向社会公布。养老托育机构对依法登记、备案承诺、履约服务、质量安全、应急管理、消防安全等承担主体责任。健全行业自律规约，加强正面宣传引导和社会舆论监督，加快构建以信用为基础的新型监管机制。
　　（二十）切实防范各类风险。加强突发事件应对，建立完善养老托育机构突发事件预防与应急准备、监测与预警、应急处置与救援、事后恢复与重建等工作机制。将养老托育纳入公共安全重点保障范围，支持服务机构安全平稳运转。完善退出机制，建立机构关停等特殊情况应急处置机制。严防“一老一小”领域以虚假投资、欺诈销售、高额返利等方式进行的非法集资，保护消费者合法权益。
　　（二十一）优化政务服务环境。完善机构设立办事指南，优化办事流程，实施并联服务，明确办理时限，推进“马上办、网上办、就近办”。制定养老托育政务服务事项清单，推进同一事项无差别受理、同标准办理，力争实现“最多跑一次”。推进养老托育政务服务的“好差评”工作，完善评价规则，加强评价结果运用，改进提升政务服务质量。
　　（二十二）积极发挥多方合力。支持公益慈善类社会组织参与，鼓励机构开发志愿服务项目，建立健全“一老一小”志愿服务项目库。引导互联网平台等社会力量建立养老托育机构用户评价体系。以普惠为导向建立多元主体参与的养老和托育产业合作平台，在要素配置、行业自律、质量安全、国际合作等方面积极作为。发挥行业协会商会等社会组织积极性，开展机构服务能力综合评价，引领行业规范发展，更好弘扬尊老爱幼社会风尚。
　　（二十三）强化数据资源支撑。依据[养老产业统计分类](https://www.pkulaw.com/chl/4a085caaee00151abdfb.html)，开展养老产业认定方法研究，推进重要指标年度统计。探索构建托育服务统计指标体系。利用智库和第三方力量加强研究，开展人口趋势预测和养老托育产业前景展望，通过发布年度报告、白皮书等形式，服务产业发展，引导社会预期。
　　坚持党委领导、政府主导，地方各级政府要建立健全“一老一小”工作推进机制，结合实际落实本意见要求，以健全政策体系、扩大服务供给、打造发展环境、完善监管服务为着力点，促进养老托育健康发展，定期向同级人民代表大会常务委员会报告服务能力提升成效。国务院各部门要根据职责分工，制定具体落实举措，推动各项任务落地。国家发展改革委要建立“一老一小”服务能力评价机制，加强对本意见落实工作的跟踪督促，及时向国务院报告。
　　附件：促进养老托育服务健康发展重点任务分工表

国务院办公厅
2020年12月14日

　　附件

　　促进养老托育服务健康发展重点任务分工表

| 序号 | 重点任务 | 责任单位 |
| --- | --- | --- |
| 1 | 根据“一老一小”人口分布和结构变化，科学谋划“十四五”养老托育服务体系，促进服务能力提质扩容和区域均衡布局。 | 民政部、国家卫生健康委、国家发展改革委、财政部、住房城乡建设部、中国残联按职责分工负责，地方各级人民政府负责。 |
| 2 | 统筹推进城乡养老托育发展。 | 民政部、国家卫生健康委、国家发展改革委按职责分工负责，地方各级人民政府负责。 |
| 3 | 积极支持普惠性服务发展。 | 国家发展改革委、民政部、国家卫生健康委按职责分工负责，地方各级人民政府负责。 |
| 4 | 在年度建设用地供应计划中保障养老托育用地需求，并结合实际安排在合理区位。 | 自然资源部、民政部、国家卫生健康委、住房城乡建设部按职责分工负责，地方各级人民政府负责。 |
| 5 | 在城市居住社区建设补短板和城镇老旧小区改造中统筹推进养老托育服务设施建设。探索允许空置公租房免费提供给社会力量供其在社区为老年人开展助餐助行、日间照料、康复护理、老年教育等服务。 | 住房城乡建设部牵头，中直管理局、国家发展改革委、民政部、自然资源部、国家卫生健康委、应急部、国务院国资委、国管局参加。 |
| 6 | 支持将各类房屋和设施用于发展养老托育，鼓励适当放宽最长租赁期限。 | 民政部、国家卫生健康委、国家发展改革委、自然资源部、住房城乡建设部、应急部按职责分工负责，地方各级人民政府负责。 |
| 7 | 非独立场所按照相关安全标准改造建设托育点并通过验收的，不需变更土地和房屋性质。 | 国家卫生健康委、自然资源部、住房城乡建设部、应急部按职责分工负责，地方各级人民政府负责。 |
| 8 | 推动财税支持政策落地。 | 财政部、税务总局、民政部、国家卫生健康委、人力资源社会保障部按职责分工负责，地方各级人民政府负责。 |
| 9 | 提高人才要素供给能力。 | 教育部、人力资源社会保障部、国家发展改革委、民政部、商务部、国家卫生健康委按职责分工负责。 |
| 10 | 增强家庭照护能力。 | 民政部、国家卫生健康委、全国妇联按职责分工负责。 |
| 11 | 研究出台家庭托育点管理办法。 | 国家卫生健康委牵头，国家发展改革委、民政部、住房城乡建设部、应急部、市场监管总局参加。 |
| 12 | 加强公办和公建民营养老机构建设，建立入住综合评估制度。 | 民政部、国家发展改革委、财政部、国家卫生健康委按职责分工负责。 |
| 13 | 完善公建民营机制，引进养老托育运营机构早期介入、全程参与项目工程建设，探索开展连锁化运营。 | 民政部、国家卫生健康委、国家发展改革委按职责分工负责。 |
| 14 | 推动培训疗养资源转型发展养老服务。 | 国家发展改革委牵头，中直管理局、民政部、财政部、人力资源社会保障部、自然资源部、住房城乡建设部、国家卫生健康委、应急部、人民银行、国务院国资委、国管局参加。 |
| 15 | 实施普惠养老托育专项行动，建设一批普惠性养老服务机构和托育服务机构。 | 国家发展改革委牵头，民政部、自然资源部、住房城乡建设部、国家卫生健康委、国务院国资委参加。 |
| 16 | 引导金融机构提升服务质效。 | 人民银行、银保监会、国家发展改革委、财政部、住房城乡建设部、证监会按职责分工负责。 |
| 17 | 促进康养融合发展。 | 国家发展改革委牵头，教育部、民政部、住房城乡建设部、文化和旅游部、国家卫生健康委、体育总局参加。 |
| 18 | 开展社区医养结合能力提升行动。 | 国家卫生健康委牵头，国家发展改革委、民政部、住房城乡建设部、国家医保局参加；地方各级人民政府负责。 |
| 19 | 提升养老机构应急保障能力。 | 民政部、国家发展改革委、应急部按职责分工负责，地方各级人民政府负责。 |
| 20 | 促进用品制造提质升级，逐步完善养老托育服务和相关用品标准体系。 | 工业和信息化部、科技部、国家发展改革委、民政部、国家卫生健康委、市场监管总局、中国残联按职责分工负责。 |
| 21 | 培育托育服务、乳粉奶业、动画设计与制作等行业民族品牌。 | 国家卫生健康委牵头，国家发展改革委、教育部、工业和信息化部、文化和旅游部、市场监管总局参加。 |
| 22 | 推进智能服务机器人后发赶超，启动康复辅助器具应用推广工程，实施智慧老龄化技术推广应用工程。 | 民政部、工业和信息化部、科技部、国家发展改革委、市场监管总局、中国残联按职责分工负责。 |
| 23 | 鼓励国内外多方共建养老托育产业合作园区，加强市场、规则、标准方面的软联通，打造制造业创新示范高地。 | 国家发展改革委牵头，工业和信息化部、民政部、人力资源社会保障部、自然资源部、国家卫生健康委、人民银行、市场监管总局参加。 |
| 24 | 加强宜居环境建设。 | 住房城乡建设部、国家卫生健康委、交通运输部、民政部、中国残联按职责分工负责。 |
| 25 | 以满足老年人生活需求和营造婴幼儿成长环境为导向，推动形成一批具有示范意义的活力发展城市和社区。 | 国家发展改革委、住房城乡建设部牵头，民政部、国家卫生健康委参加。 |
| 26 | 完善养老托育服务综合监管体系。 | 民政部、国家卫生健康委、市场监管总局、住房城乡建设部、应急部按职责分工负责。 |
| 27 | 将养老托育纳入公共安全重点保障范围。 | 民政部、国家卫生健康委、国家发展改革委、应急部、市场监管总局按职责分工负责，地方各级人民政府负责。 |
| 28 | 严防“一老一小”领域非法集资。 | 民政部、国家卫生健康委、银保监会按职责分工负责，地方各级人民政府负责。 |
| 29 | 制定养老托育政务服务事项清单，推进养老托育政务服务的“好差评”工作。 | 民政部、国家卫生健康委、市场监管总局按职责分工负责，地方各级人民政府负责。 |
| 30 | 支持公益慈善类社会组织参与，建立健全“一老一小”志愿服务项目库。 | 全国总工会、共青团中央、中国残联、民政部、国家卫生健康委按职责分工负责。 |
| 31 | 以普惠为导向建立多元主体参与的养老和托育产业合作平台。 | 国家发展改革委牵头，工业和信息化部、民政部、自然资源部、住房城乡建设部、文化和旅游部、国家卫生健康委、国务院国资委、市场监管总局参加。 |
| 32 | 依据[养老产业统计分类](https://www.pkulaw.com/chl/4a085caaee00151abdfb.html)，开展养老产业认定方法研究，推进重要指标年度统计，探索构建托育服务统计指标体系。 | 国家统计局牵头，国家发展改革委、工业和信息化部、民政部、住房城乡建设部、国家卫生健康委、市场监管总局、中国残联参加。 |
| 33 | 建立“一老一小”服务能力评价机制，加强对本意见执行情况的跟踪督促。 | 国家发展改革委牵头，各相关部门参加。 |

© 北大法宝：（[www.pkulaw.com](https://www.pkulaw.com/)) 专业提供法律信息、法学知识和法律软件领域各类解决方案。北大法宝为您提供丰富的参考资料，正式引用法规条文时请与标准文本核对。
欢迎查看所有[产品和服务](https://www.pkulaw.com/helps/2.html)。法宝快讯：[如何快速找到您需要的检索结果？](https://www.pkulaw.com/helps/70.html)[法宝](https://www.pkulaw.com/helps/69.html)V6有何新特色？

扫描二维码阅读原文

**原文链接：**<https://www.pkulaw.com/chl/d1ad8fb179db9915bdfb.html>
